# Rectosigmoid Adenocarcinoma Presenting As Nine Days of Constipation

**DOI:** 10.7759/cureus.38393

**Published:** 2023-05-01

**Authors:** Sri Sai Prasanna Thota, Jayani Senanayake, Shubhneet Gill, Sarah Craig, Timothy Stear

**Affiliations:** 1 Medicine, Washington University of Health and Science, Chicago, USA; 2 Medicine, Saint James School of Medicine, The Quarter, AIA; 3 Medicine, American University School of Medicine, Suwanee, USA; 4 General Surgery, Community First Medical Center, Chicago, USA

**Keywords:** colon resection, gi oncology, tubulovillous adenocarcinoma, screening colonoscopy, surgery general, rectosigmoid mass, functional constipation, rectosigmoid resection, sigmoid adenocarcinoma, colon cancer prevention

## Abstract

We report the case of a 56-year-old male presenting with nine days of constipation and absence of flatus without any improvement and who had received conservative management after recent admission at an external hospital. Upon further investigation, the patient was diagnosed with rectosigmoid adenocarcinoma and was successfully surgically treated without any perioperative complications. This case highlights the importance of early detection and interventions necessary to prevent progression of colorectal adenocarcinoma. Easily manageable symptoms such as constipation may require further evaluation by implementing a constipation scoring system to avoid missed diagnoses such as cancer and metastasis. Therefore, the association between constipation and colorectal carcinoma warrants further research investigations as well as clinician awareness to prevent life-threatening complications.

## Introduction

Currently, the second deadliest malignancy and third most commonly diagnosed cancer in both males and females is colorectal cancer [1]. Most colorectal cancers are carcinomas with adenocarcinoma constituting 90% of them. An increased risk of neoplasia presents with villous and tubulovillous histology compared to tubular adenomas, and an increased risk of malignant transformation is associated with high-grade dysplasia [2]. Early detection directly correlates with a favorable prognosis. From 1975 to 2014, a 51% decrease in mortality due to colorectal cancer in the United States has been accredited to early detection [3]. Colorectal carcinoma can present as sporadic, familial, and inherited syndromes with the sporadic form being the most common [1]. The associated symptoms with this type of carcinoma have been reported as changes in bowel habits, such as diarrhea and/or constipation, inconsistency of stool, blood in the stool, and rectal bleeding. Abdominal pain and discomfort with the accumulation of gas along with cramping are also possible symptoms of colorectal cancer [4]. 

Detection of colorectal cancer is done through a series of tests and examinations. A routine colonoscopy is recommended beginning at age 45 to detect any polyps or abnormal masses. Resection or biopsy of the polyp or mass is performed for pathologic examination for the presence or absence of cancer [4]. Stool tests are used to identify blood and tumor markers. Anemia due to bleeding and the presence of elevated carcinoembryonic antigen (CEA) can be determined through serum testing. Computed tomography and MRI can be conducted to evaluate for malignancy and the possibility of metastasis [5]. According to the American Joint Committee, colorectal cancer is staged based on the tumor size and depth of invasion, spread to the lymph nodes, and metastasis to other organs, otherwise known as the tumor, nodes and metastases (TNM) staging system. Combined, colorectal cancer is staged from 0-4 with stage 0 being the earliest stage and stage 4 being the most severe form of colorectal cancer. In addition, colorectal cancer can also be described by its grade (G) based on microscopic changes. While G1 indicates a “well-differentiated” tumor, G4 indicates an “undifferentiated” tumor grade with a worse prognosis [6].

Standard treatment of colorectal cancer includes surgical or endoscopic resection, immunotherapy, chemotherapy, radiation therapy, and/or targeted therapy using monoclonal antibodies [7]. Along with the current diagnostic measures in place, employing constipation scoring guidelines can help with the detection and management of occult carcinomas. We present the case of a 56-year-old male with an undetected sigmoid and rectal adenocarcinoma who initially presented to the hospital with nine days of constipation and was ultimately treated with low anterior colon resection and end colostomy creation.

## Case presentation

A 56-year-old male presented to the emergency department (ED) with abdominal pain, constipation, and lack of bowel movement for nine days. The patient had a past medical history significant for alopecia and gastroesophageal reflux disease (GERD). His family history and social history are non-contributory. On the primary survey, the patient’s abdomen was largely distended and non-tender. Bowel sounds were active in all four quadrants with no palpable masses, organomegaly, or peritoneal signs. The patient reported prior issues with constipation, but no previous episode was of this duration. Of note, the patient did not report any other change in bowel habits such as diarrhea, narrowing of the stool, or rectal bleeding. At this time, the patient reported compliance with his outpatient medications prescribed for constipation, which included senna-docusate, magnesium citrate, polyethylene glycol, and fiber supplements. Notably, the patient denied prior colorectal cancer screening with colonoscopy or sigmoidoscopy. In the ED, he was initially treated conservatively with a nasogastric tube placement, and nothing by mouth (NPO). The patient was afebrile and his vital signs were stable. Laboratory investigations performed in the ED are listed in Table 1. 

**Table 1:** Laboratory workup performed at the emergency department

Test	Result	Reference range
Hemoglobin	10.2 g/dL	13.0- 17.0 g/dL
Hematocrit	33.8 %	38.6- 49.2%
White blood cells	8.6 k/mm cu	4.0- 11.0 k/mm cu
Platelets	751 k/mm cu	150- 450 k/mm cu
Mean corpuscular volume	67.8 fL	80.0- 100.0 fL
Mean corpuscular hemoglobin	20.5 pg	26.0- 34.0 pg
Mean corpuscular hemoglobin concentration	30.3 %	32.5- 35.8%
Red cell distribution width	18.1 %	11.9- 15.9%
Carcinoembryonic Antigen (CEA)	13 ng/mL	0.0- 2.5 ng/mL

**Figure 1:**
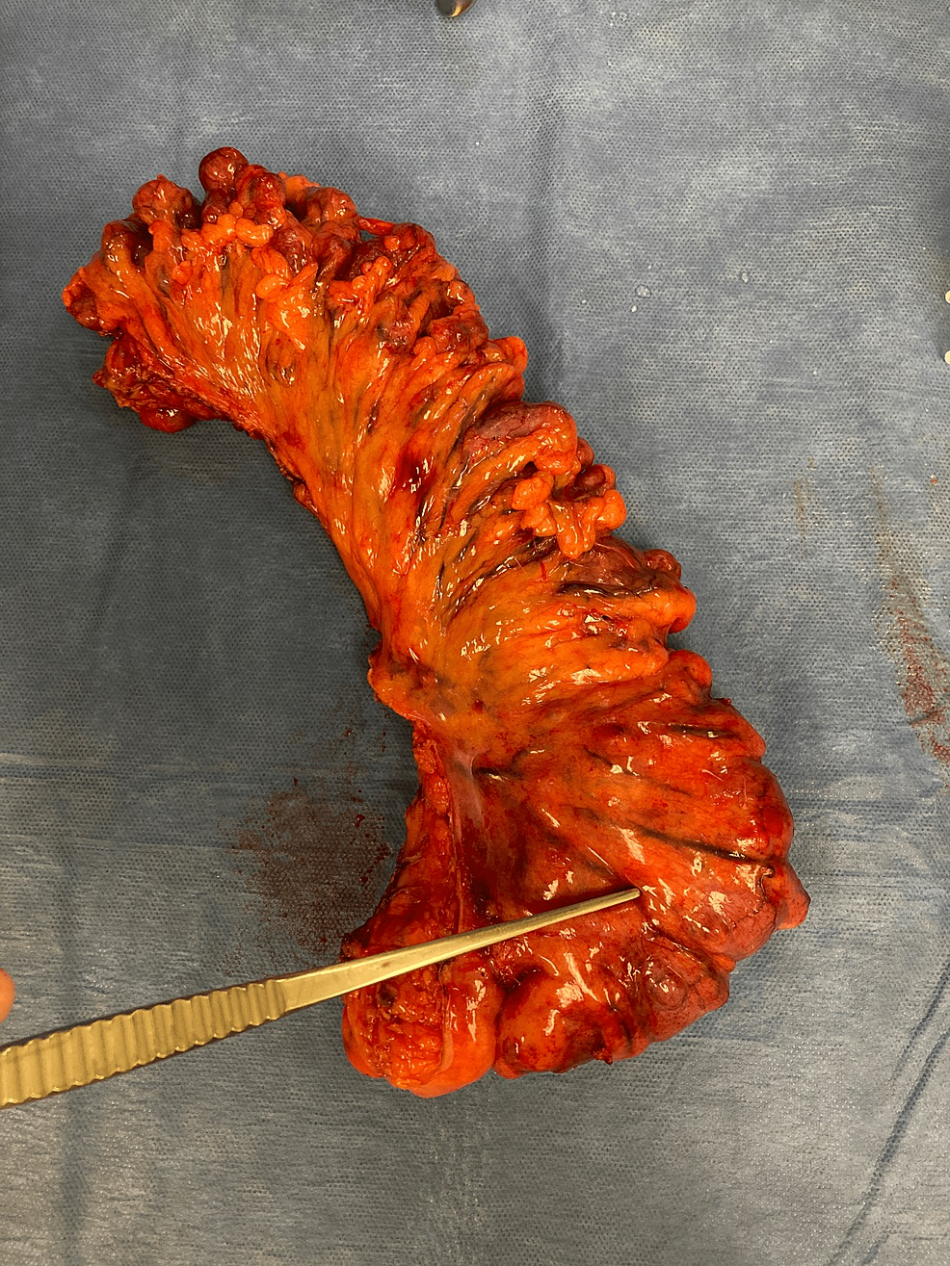
Gross specimen showing a mass in the rectosigmoid colon along with diverticulosis

**Figure 2:**
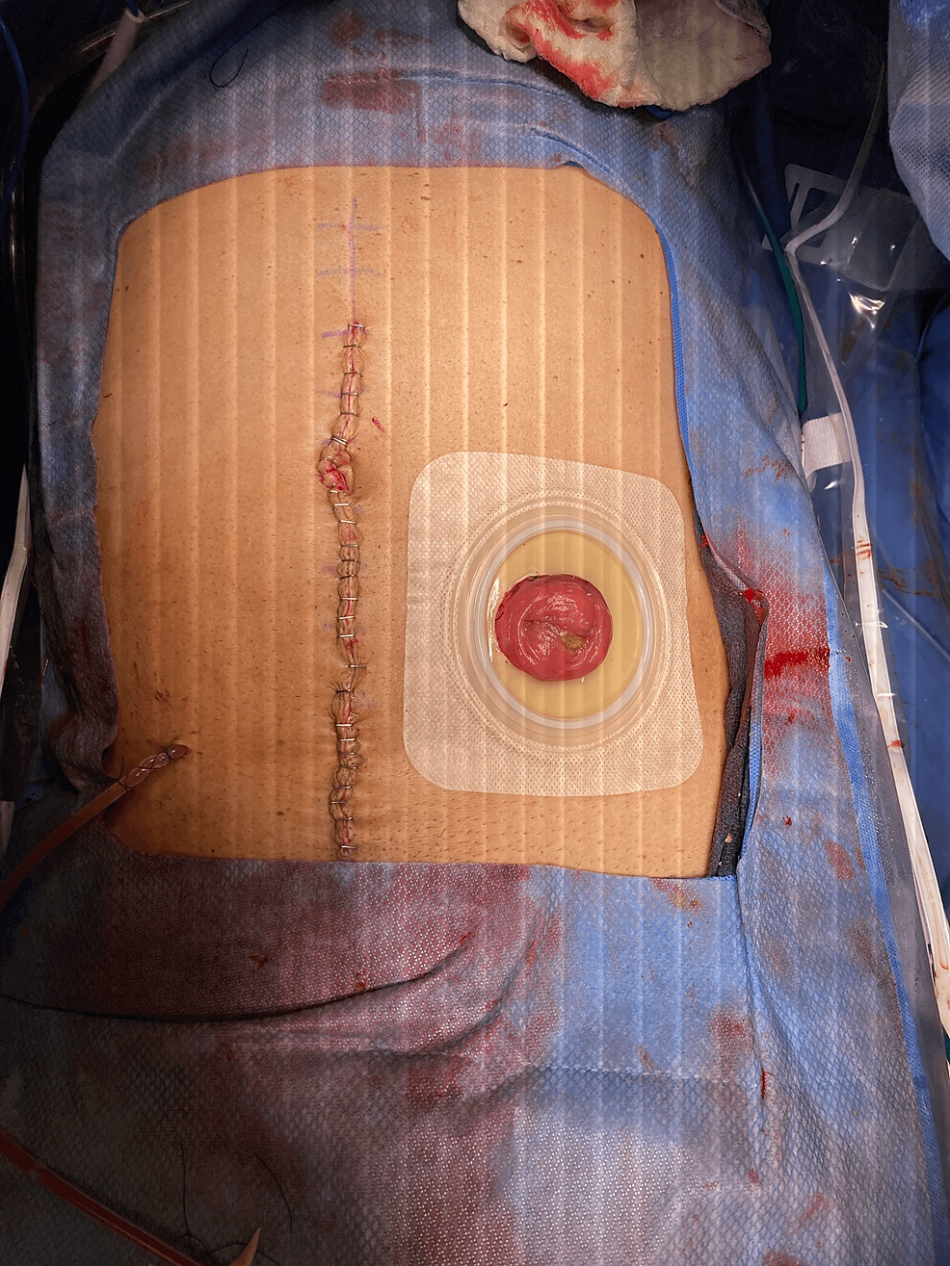
Postoperative incision showing closure with staples, placement of Jackson-Pratt drain, and colostomy

The patient was admitted to the hospital soon after and imaging studies were ordered to further investigate his symptoms of constipation. Computed tomography scan of the abdomen and pelvis raised suspicion for an underlying mass or irregular wall thickening at the rectosigmoid junction. A fluoroscopy of the colon without air contrast was then ordered, which demonstrated moderate stenosis and a filling defect of the distal sigmoid colon suspicious for malignancy. A sigmoidoscopy was performed next, revealing a likely malignant, obstructing tumor in the rectosigmoid colon. Surgery was consulted thereafter and the patient was scheduled for a sigmoid tumor resection with colostomy placement. During the procedure, a large mass was palpated in the rectosigmoid junction which was removed. The specimen included the tumor and rectosigmoid colon which was sent to pathology (Figure 1). Additionally, scattered large diverticula were noted to be present throughout the sigmoid colon (also seen in Figure 1). The abdomen was further inspected and revealed no signs of metastatic disease, liver nodules, or peritoneal implants. The patient’s abdomen was extensively irrigated out with warm saline irrigation to reduce post-surgical site infections, hemostasis was achieved with electrocautery, a Jackson-Pratt (JP) drain was placed, an end ostomy was created due to the inability to tolerate a bowel preparation, and the incision was closed with staples (Figure 2). The patient tolerated the procedure well without any intraoperative complications. A postoperative chest CT showed no evidence of metastatic disease.

**Figure 3:**
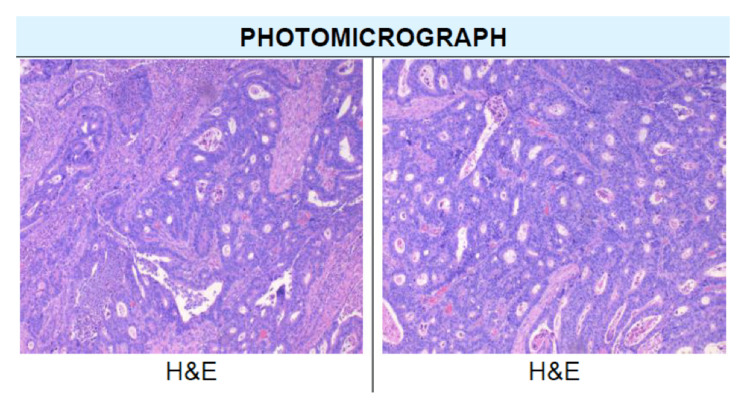
Hematoxylin and eosin (H&E) stained sigmoid colon showing grade two, moderately differentiated adenocarcinoma

The pathology report results indicated a 6.0 cm invasive, grade two, moderately differentiated adenocarcinoma in the sigmoid colon and rectum (Figure 3). The tumor arose in a tubulovillous adenoma polyp and invaded through the muscularis propria into the pericolorectal tissues without lymphovascular invasion or perineural invasion. All margins were negative for invasive carcinoma. Additionally, a total of 17 lymph nodes were identified which were all without signs of metastasis. An immunohistochemical study of DNA mismatch repair demonstrated that all four proteins (MLH 1, MSH 2, MSH 5, PMS 2) were intact. Immunostain for HER-2 and PD-L1 yielded negative results. The patient was discharged on postoperative day three without any perioperative complications. The patient followed up one week postoperatively for drain removal and again at two weeks postoperatively for staple removal. He has not had any readmissions at the time of writing and was advised to follow up with his primary care physician. 

## Discussion

Referral of patients with unremitting constipation to a gastroenterologist or surgeon should be performed for early detection of occult colorectal cancer as a cause of constipation. The majority of sigmoid and rectal cancers present with vague symptoms such as rectal bleeding, bowel habit changes, or constipation [8]. Although constipation does not pose as a high-risk clinical symptom by recent guidelines, it is known to be a common symptom among patients with sigmoid and rectal carcinomas [9]. In this present case report, we investigated the association between constipation, presenting as the primary symptom, and the diagnosis of sigmoid colon and rectal adenocarcinoma. A study was performed to assess a standard scoring system for the evaluation of constipated patients and found that 22.3% of the patients evaluated had a constipation score of greater than eight, which is similar to the prevalence of constipation present in patients found to have colorectal carcinoma [10]. It is suggested, in this case, that qualitative and quantitative assessment of the severity of constipation could provide a more beneficial overview of the patient in regard to testing for sigmoid and rectal carcinomas.

Our patient presented with nine days of constipation as a consequence of previously undetected sigmoid and rectal adenocarcinoma. A previously published Australian report from 2019 describes the case of a 36-year-old woman who was 16 weeks pregnant and presenting with chronic constipation over two weeks in which a pathology study revealed an invasive, mucinous adenocarcinoma in the lower rectum [11]. Along with the chronic constipation, the case describes the patient having cessation of the elimination of gas and feces, abdominal distention, diffuse abdominal pain, and diffuse distention of the colon upon MRI of the pelvis. Another study detailed the case of a 34-year-old man with recent onset constipation who presented with colonic obstruction due to a palpable rectal tumor, which was then resected and was noted to originate at the rectosigmoid junction [12]. In the majority of the above cases, the primary complaint of constipation led to the eventual diagnosis of sigmoid and rectal carcinoma. Relative to other presenting symptoms, prolonged constipation, although rare, is a potentially life-threatening complication. Thus, it is imperative that early detection systems and surgical interventions be implemented in such patients to detect the presence of obstructing colorectal carcinomas.

Due to our patient’s presentation, CT and colonoscopy were the initial management measures taken in order to ultimately diagnose an obstructing colorectal adenocarcinoma, which was initially thought to be constipation. Diagnostic imaging with findings concerning colorectal mass led to rectosigmoid resection with end colostomy creation. The procedure was successfully executed without complications. Prompt surgical intervention was pivotal in the prevention of complications such as bowel perforation. The development of constipation scoring guidelines and prompt imaging or endoscopic evaluation may aid in the detection of such carcinomas. Clinician awareness of the possibility of occult colorectal carcinoma in correlation with the patient’s history and symptoms may guide the path for a comprehensive review and treatment course. By highlighting this connection, our case intends to further awareness for improved clinical outcomes and future opportunities for research.

## Conclusions

Although chronic constipation is common, it has been noted to be a sensitive indicator in multiple cases of colorectal cancer, even being the primary presenting symptom for many. Although constipation can be multifactorial, it can become life-threatening and therefore early interventions should be implemented. Colorectal cancer is the second leading cause of malignancy and has a favorable outcome if identified early. Along with regular screening, a better understanding of commonly presenting symptom patterns would aid in earlier detection. This report intends to acquaint clinical awareness with the patient’s history and presenting symptoms.
